# Supplementary material for: Modeling glioblastoma heterogeneity as a dynamic network of cell states
Source: Mol Syst Biol. 2021 Sep 16;17(9):e10105. doi: 10.15252/msb.202010105 (PMC8444284; doi:10.15252/msb.202010105)
Supplement: Supplementary file 5 — Source Data for Figure 3 [file MSB-17-e10105-s001.zip › Figure3A_sourcedata/GSEA_3065/nosigdb_state3.GseaPreranked.1622718426028/RICHARDS_NOWAKOWSKI_SCIENCE_2017_OPC_UPREG.html]

Details for gene set RICHARDS\_NOWAKOWSKI\_SCIENCE\_2017\_OPC\_UPREG[GSEA]

|  || Dataset | state3 |
| Phenotype | NoPhenotypeAvailable |
| Upregulated in class | na\_pos |
| GeneSet | RICHARDS\_NOWAKOWSKI\_SCIENCE\_2017\_OPC\_UPREG |
| Enrichment Score (ES) | 0.5661385 |
| Normalized Enrichment Score (NES) | 2.537557 |
| Nominal p-value | 0.0 |
| FDR q-value | 0.0 |
| FWER p-Value | 0.0 |
Table: GSEA Results Summary

  

Fig 1: Enrichment plot: RICHARDS\_NOWAKOWSKI\_SCIENCE\_2017\_OPC\_UPREG      
 Profile of the Running ES Score & Positions of GeneSet Members on the Rank Ordered List

  

| PROBE | GENE SYMBOL | GENE\_TITLE | RANK IN GENE LIST | RANK METRIC SCORE | RUNNING ES | CORE ENRICHMENT || 1 | BCAN |  |  | 0 | 0.883 | 0.0179 | Yes |
| 2 | SCRG1 |  |  | 3 | 0.645 | 0.0307 | Yes |
| 3 | HSP90B1 |  |  | 7 | 0.563 | 0.0418 | Yes |
| 4 | PDIA6 |  |  | 8 | 0.549 | 0.0529 | Yes |
| 5 | B2M |  |  | 11 | 0.538 | 0.0636 | Yes |
| 6 | PTPRZ1 |  |  | 14 | 0.532 | 0.0741 | Yes |
| 7 | PLAT |  |  | 18 | 0.513 | 0.0842 | Yes |
| 8 | PTN |  |  | 22 | 0.493 | 0.0938 | Yes |
| 9 | CANX |  |  | 27 | 0.479 | 0.1031 | Yes |
| 10 | PGRMC1 |  |  | 29 | 0.473 | 0.1125 | Yes |
| 11 | LAPTM4B |  |  | 31 | 0.473 | 0.1220 | Yes |
| 12 | CD9 |  |  | 32 | 0.473 | 0.1316 | Yes |
| 13 | ABHD2 |  |  | 36 | 0.467 | 0.1407 | Yes |
| 14 | GPM6A |  |  | 37 | 0.460 | 0.1500 | Yes |
| 15 | GRIA2 |  |  | 38 | 0.460 | 0.1593 | Yes |
| 16 | HSPA5 |  |  | 43 | 0.447 | 0.1679 | Yes |
| 17 | ALCAM |  |  | 45 | 0.440 | 0.1767 | Yes |
| 18 | FGFBP3 |  |  | 51 | 0.431 | 0.1849 | Yes |
| 19 | DSEL |  |  | 62 | 0.392 | 0.1916 | Yes |
| 20 | PON2 |  |  | 63 | 0.389 | 0.1995 | Yes |
| 21 | SPCS3 |  |  | 76 | 0.370 | 0.2056 | Yes |
| 22 | DST |  |  | 80 | 0.365 | 0.2126 | Yes |
| 23 | ITM2B |  |  | 82 | 0.361 | 0.2198 | Yes |
| 24 | SLC44A1 |  |  | 83 | 0.361 | 0.2271 | Yes |
| 25 | SOX6 |  |  | 88 | 0.358 | 0.2339 | Yes |
| 26 | MMP16 |  |  | 94 | 0.351 | 0.2404 | Yes |
| 27 | KCNQ1OT1 |  |  | 99 | 0.347 | 0.2470 | Yes |
| 28 | GPM6B |  |  | 105 | 0.343 | 0.2534 | Yes |
| 29 | SCD |  |  | 108 | 0.340 | 0.2600 | Yes |
| 30 | PDGFRA |  |  | 115 | 0.326 | 0.2659 | Yes |
| 31 | SEMA5A |  |  | 121 | 0.324 | 0.2719 | Yes |
| 32 | TMX3 |  |  | 123 | 0.322 | 0.2783 | Yes |
| 33 | SCARB2 |  |  | 132 | 0.318 | 0.2838 | Yes |
| 34 | BCHE |  |  | 136 | 0.315 | 0.2898 | Yes |
| 35 | TJP1 |  |  | 139 | 0.314 | 0.2959 | Yes |
| 36 | PPIB |  |  | 142 | 0.313 | 0.3021 | Yes |
| 37 | PCDH9 |  |  | 147 | 0.310 | 0.3079 | Yes |
| 38 | LRP1 |  |  | 152 | 0.309 | 0.3137 | Yes |
| 39 | ILDR2 |  |  | 171 | 0.299 | 0.3176 | Yes |
| 40 | CHD7 |  |  | 172 | 0.298 | 0.3236 | Yes |
| 41 | SAR1B |  |  | 195 | 0.287 | 0.3268 | Yes |
| 42 | YIPF6 |  |  | 200 | 0.283 | 0.3321 | Yes |
| 43 | MYO10 |  |  | 206 | 0.280 | 0.3372 | Yes |
| 44 | DAG1 |  |  | 209 | 0.278 | 0.3426 | Yes |
| 45 | RDH11 |  |  | 212 | 0.277 | 0.3480 | Yes |
| 46 | SEPT7 |  |  | 221 | 0.274 | 0.3526 | Yes |
| 47 | PCDHGC3 |  |  | 228 | 0.272 | 0.3574 | Yes |
| 48 | TRIO |  |  | 235 | 0.269 | 0.3621 | Yes |
| 49 | DHCR24 |  |  | 244 | 0.265 | 0.3666 | Yes |
| 50 | SERINC5 |  |  | 252 | 0.263 | 0.3711 | Yes |
| 51 | SRPRB |  |  | 257 | 0.260 | 0.3759 | Yes |
| 52 | NOVA1 |  |  | 270 | 0.253 | 0.3796 | Yes |
| 53 | ITM2C |  |  | 273 | 0.252 | 0.3844 | Yes |
| 54 | VCAN |  |  | 293 | 0.245 | 0.3871 | Yes |
| 55 | CADM4 |  |  | 299 | 0.244 | 0.3915 | Yes |
| 56 | IL1RAP |  |  | 303 | 0.242 | 0.3960 | Yes |
| 57 | EDNRB |  |  | 305 | 0.242 | 0.4008 | Yes |
| 58 | LSAMP |  |  | 307 | 0.241 | 0.4056 | Yes |
| 59 | SERPINE2 |  |  | 308 | 0.241 | 0.4105 | Yes |
| 60 | SALL3 |  |  | 312 | 0.240 | 0.4150 | Yes |
| 61 | IL6ST |  |  | 316 | 0.239 | 0.4195 | Yes |
| 62 | ITGAV |  |  | 322 | 0.238 | 0.4237 | Yes |
| 63 | THY1 |  |  | 326 | 0.236 | 0.4281 | Yes |
| 64 | PRKCA |  |  | 328 | 0.234 | 0.4328 | Yes |
| 65 | NTRK2 |  |  | 336 | 0.233 | 0.4367 | Yes |
| 66 | MPZL1 |  |  | 340 | 0.231 | 0.4410 | Yes |
| 67 | MAP2 |  |  | 344 | 0.231 | 0.4453 | Yes |
| 68 | COPB2 |  |  | 353 | 0.229 | 0.4490 | Yes |
| 69 | MRPL32 |  |  | 367 | 0.227 | 0.4521 | Yes |
| 70 | DOCK10 |  |  | 395 | 0.219 | 0.4533 | Yes |
| 71 | CADM2 |  |  | 416 | 0.215 | 0.4553 | Yes |
| 72 | HSD17B12 |  |  | 432 | 0.212 | 0.4578 | Yes |
| 73 | EMC10 |  |  | 433 | 0.212 | 0.4621 | Yes |
| 74 | PLEKHH2 |  |  | 440 | 0.211 | 0.4657 | Yes |
| 75 | ACTN4 |  |  | 449 | 0.210 | 0.4690 | Yes |
| 76 | ITGB8 |  |  | 463 | 0.206 | 0.4717 | Yes |
| 77 | MAGI1 |  |  | 476 | 0.202 | 0.4743 | Yes |
| 78 | UFL1 |  |  | 492 | 0.200 | 0.4766 | Yes |
| 79 | TM7SF3 |  |  | 495 | 0.199 | 0.4804 | Yes |
| 80 | LAMA4 |  |  | 496 | 0.199 | 0.4844 | Yes |
| 81 | LRRC4C |  |  | 509 | 0.197 | 0.4870 | Yes |
| 82 | NT5E |  |  | 511 | 0.197 | 0.4909 | Yes |
| 83 | DPP6 |  |  | 534 | 0.194 | 0.4922 | Yes |
| 84 | SDC3 |  |  | 539 | 0.194 | 0.4957 | Yes |
| 85 | HIPK2 |  |  | 545 | 0.193 | 0.4990 | Yes |
| 86 | KIZ |  |  | 554 | 0.192 | 0.5019 | Yes |
| 87 | NLGN3 |  |  | 558 | 0.191 | 0.5055 | Yes |
| 88 | LRRN3 |  |  | 574 | 0.189 | 0.5075 | Yes |
| 89 | EPB41L2 |  |  | 596 | 0.187 | 0.5088 | Yes |
| 90 | OLIG1 |  |  | 602 | 0.186 | 0.5120 | Yes |
| 91 | HAS2 |  |  | 603 | 0.186 | 0.5157 | Yes |
| 92 | PHLDB1 |  |  | 606 | 0.185 | 0.5193 | Yes |
| 93 | SCD5 |  |  | 619 | 0.184 | 0.5216 | Yes |
| 94 | LIMA1 |  |  | 639 | 0.180 | 0.5230 | Yes |
| 95 | CASK |  |  | 643 | 0.180 | 0.5263 | Yes |
| 96 | PLOD2 |  |  | 683 | 0.173 | 0.5252 | Yes |
| 97 | COL11A1 |  |  | 684 | 0.173 | 0.5287 | Yes |
| 98 | NTRK3 |  |  | 687 | 0.173 | 0.5319 | Yes |
| 99 | AASS |  |  | 696 | 0.170 | 0.5344 | Yes |
| 100 | SLC35F1 |  |  | 711 | 0.168 | 0.5362 | Yes |
| 101 | CSPG5 |  |  | 716 | 0.168 | 0.5391 | Yes |
| 102 | P2RX7 |  |  | 723 | 0.167 | 0.5418 | Yes |
| 103 | HIP1 |  |  | 728 | 0.167 | 0.5447 | Yes |
| 104 | ANGPTL2 |  |  | 758 | 0.162 | 0.5446 | Yes |
| 105 | CLDND1 |  |  | 773 | 0.160 | 0.5462 | Yes |
| 106 | DNM3 |  |  | 783 | 0.159 | 0.5483 | Yes |
| 107 | BLM |  |  | 811 | 0.156 | 0.5483 | Yes |
| 108 | PHLPP1 |  |  | 819 | 0.156 | 0.5506 | Yes |
| 109 | TNK2 |  |  | 839 | 0.154 | 0.5515 | Yes |
| 110 | NAPEPLD |  |  | 847 | 0.153 | 0.5538 | Yes |
| 111 | SSR1 |  |  | 851 | 0.152 | 0.5565 | Yes |
| 112 | GOLIM4 |  |  | 892 | 0.149 | 0.5548 | Yes |
| 113 | ERBB3 |  |  | 899 | 0.149 | 0.5571 | Yes |
| 114 | NOC3L |  |  | 939 | 0.144 | 0.5554 | Yes |
| 115 | LRP1B |  |  | 953 | 0.142 | 0.5567 | Yes |
| 116 | ETV1 |  |  | 963 | 0.141 | 0.5585 | Yes |
| 117 | LEPROT |  |  | 974 | 0.140 | 0.5602 | Yes |
| 118 | WSCD1 |  |  | 978 | 0.140 | 0.5626 | Yes |
| 119 | TMX1 |  |  | 1053 | 0.134 | 0.5566 | Yes |
| 120 | PCDH17 |  |  | 1059 | 0.134 | 0.5587 | Yes |
| 121 | GPR155 |  |  | 1095 | 0.130 | 0.5572 | Yes |
| 122 | APOD |  |  | 1113 | 0.129 | 0.5578 | Yes |
| 123 | ITPR2 |  |  | 1114 | 0.129 | 0.5604 | Yes |
| 124 | PGM3 |  |  | 1133 | 0.127 | 0.5609 | Yes |
| 125 | SETD5 |  |  | 1164 | 0.125 | 0.5598 | Yes |
| 126 | TMEM132B |  |  | 1194 | 0.122 | 0.5589 | Yes |
| 127 | CSGALNACT1 |  |  | 1202 | 0.121 | 0.5605 | Yes |
| 128 | MAP3K1 |  |  | 1219 | 0.120 | 0.5610 | Yes |
| 129 | LRRK2 |  |  | 1229 | 0.119 | 0.5624 | Yes |
| 130 | SULF2 |  |  | 1232 | 0.119 | 0.5646 | Yes |
| 131 | KIAA0232 |  |  | 1240 | 0.119 | 0.5661 | Yes |
| 132 | SLC2A13 |  |  | 1274 | 0.117 | 0.5646 | No |
| 133 | ATCAY |  |  | 1327 | 0.113 | 0.5607 | No |
| 134 | CHRM3 |  |  | 1370 | 0.110 | 0.5580 | No |
| 135 | PTPRE |  |  | 1396 | 0.109 | 0.5572 | No |
| 136 | KANK1 |  |  | 1398 | 0.108 | 0.5593 | No |
| 137 | SLC22A17 |  |  | 1419 | 0.107 | 0.5591 | No |
| 138 | ZNF37A |  |  | 1425 | 0.107 | 0.5607 | No |
| 139 | SPRY4 |  |  | 1461 | 0.105 | 0.5587 | No |
| 140 | SPRY1 |  |  | 1471 | 0.105 | 0.5597 | No |
| 141 | TMEM206 |  |  | 1477 | 0.105 | 0.5612 | No |
| 142 | NLGN4X |  |  | 1480 | 0.105 | 0.5631 | No |
| 143 | CTTNBP2 |  |  | 1518 | 0.102 | 0.5608 | No |
| 144 | LRRN1 |  |  | 1690 | 0.092 | 0.5424 | No |
| 145 | C1orf61 |  |  | 1703 | 0.091 | 0.5429 | No |
| 146 | CAMSAP2 |  |  | 1704 | 0.091 | 0.5447 | No |
| 147 | LPP |  |  | 1725 | 0.090 | 0.5442 | No |
| 148 | ZNF462 |  |  | 1733 | 0.090 | 0.5452 | No |
| 149 | TSPAN7 |  |  | 1738 | 0.089 | 0.5465 | No |
| 150 | OLIG2 |  |  | 1742 | 0.089 | 0.5480 | No |
| 151 | SPATA6 |  |  | 1796 | 0.087 | 0.5434 | No |
| 152 | BMP7 |  |  | 1871 | 0.082 | 0.5363 | No |
| 153 | MAML2 |  |  | 1883 | 0.082 | 0.5367 | No |
| 154 | GCC2 |  |  | 1944 | 0.078 | 0.5312 | No |
| 155 | PEAK1 |  |  | 1984 | 0.077 | 0.5281 | No |
| 156 | DSCAM |  |  | 2007 | 0.076 | 0.5270 | No |
| 157 | APBB2 |  |  | 2011 | 0.076 | 0.5282 | No |
| 158 | BCAT1 |  |  | 2034 | 0.075 | 0.5271 | No |
| 159 | PLP1 |  |  | 2047 | 0.074 | 0.5272 | No |
| 160 | SAMD8 |  |  | 2108 | 0.072 | 0.5216 | No |
| 161 | ST3GAL5 |  |  | 2139 | 0.071 | 0.5194 | No |
| 162 | EPN2 |  |  | 2164 | 0.070 | 0.5180 | No |
| 163 | RNF157 |  |  | 2181 | 0.069 | 0.5175 | No |
| 164 | GRB14 |  |  | 2251 | 0.066 | 0.5107 | No |
| 165 | KLHL42 |  |  | 2260 | 0.066 | 0.5111 | No |
| 166 | TRIB2 |  |  | 2296 | 0.065 | 0.5082 | No |
| 167 | CALCRL |  |  | 2315 | 0.064 | 0.5074 | No |
| 168 | DNER |  |  | 2341 | 0.063 | 0.5057 | No |
| 169 | RFTN2 |  |  | 2346 | 0.063 | 0.5065 | No |
| 170 | IDH1 |  |  | 2376 | 0.062 | 0.5043 | No |
| 171 | PDE4B |  |  | 2443 | 0.059 | 0.4977 | No |
| 172 | SHC3 |  |  | 2460 | 0.058 | 0.4970 | No |
| 173 | APCDD1 |  |  | 2511 | 0.057 | 0.4922 | No |
| 174 | ARHGAP42 |  |  | 2515 | 0.056 | 0.4930 | No |
| 175 | SEMA6D |  |  | 2534 | 0.056 | 0.4920 | No |
| 176 | GLDC |  |  | 2660 | 0.051 | 0.4782 | No |
| 177 | NLGN1 |  |  | 2733 | 0.049 | 0.4707 | No |
| 178 | ZEB1 |  |  | 2764 | 0.048 | 0.4681 | No |
| 179 | UGDH |  |  | 2879 | 0.045 | 0.4555 | No |
| 180 | SHISA9 |  |  | 2964 | 0.042 | 0.4464 | No |
| 181 | SFT2D1 |  |  | 3004 | 0.041 | 0.4427 | No |
| 182 | LDHB |  |  | 3031 | 0.040 | 0.4404 | No |
| 183 | ABAT |  |  | 3059 | 0.040 | 0.4380 | No |
| 184 | CPEB2 |  |  | 3071 | 0.039 | 0.4375 | No |
| 185 | REEP3 |  |  | 3147 | 0.037 | 0.4294 | No |
| 186 | NUP50 |  |  | 3160 | 0.037 | 0.4287 | No |
| 187 | IQSEC1 |  |  | 3221 | 0.035 | 0.4223 | No |
| 188 | ZADH2 |  |  | 3296 | 0.033 | 0.4142 | No |
| 189 | NTN1 |  |  | 3324 | 0.032 | 0.4117 | No |
| 190 | RNF144A |  |  | 3368 | 0.031 | 0.4072 | No |
| 191 | TNS3 |  |  | 3372 | 0.031 | 0.4075 | No |
| 192 | MED11 |  |  | 3444 | 0.029 | 0.3997 | No |
| 193 | TAOK3 |  |  | 3466 | 0.029 | 0.3978 | No |
| 194 | STXBP3 |  |  | 3477 | 0.029 | 0.3972 | No |
| 195 | FARP2 |  |  | 3537 | 0.027 | 0.3907 | No |
| 196 | E2F3 |  |  | 3553 | 0.027 | 0.3895 | No |
| 197 | TNRC6C |  |  | 3572 | 0.026 | 0.3879 | No |
| 198 | PIK3R1 |  |  | 3604 | 0.025 | 0.3847 | No |
| 199 | TAF2 |  |  | 3611 | 0.025 | 0.3845 | No |
| 200 | TENM1 |  |  | 3624 | 0.025 | 0.3836 | No |
| 201 | NXPH1 |  |  | 3773 | 0.021 | 0.3665 | No |
| 202 | ZNF516 |  |  | 3872 | 0.018 | 0.3553 | No |
| 203 | CHST3 |  |  | 3949 | 0.016 | 0.3466 | No |
| 204 | GNB4 |  |  | 3971 | 0.016 | 0.3444 | No |
| 205 | PDGFA |  |  | 3998 | 0.015 | 0.3417 | No |
| 206 | MAP4K5 |  |  | 4012 | 0.015 | 0.3404 | No |
| 207 | ETV5 |  |  | 4087 | 0.013 | 0.3319 | No |
| 208 | GLCCI1 |  |  | 4180 | 0.010 | 0.3212 | No |
| 209 | SMAD5 |  |  | 4201 | 0.010 | 0.3191 | No |
| 210 | FOXN2 |  |  | 4204 | 0.009 | 0.3190 | No |
| 211 | STRN |  |  | 4209 | 0.009 | 0.3187 | No |
| 212 | BPNT1 |  |  | 4231 | 0.009 | 0.3164 | No |
| 213 | UBE3B |  |  | 4286 | 0.007 | 0.3102 | No |
| 214 | SEPT2 |  |  | 4315 | 0.006 | 0.3070 | No |
| 215 | PDLIM5 |  |  | 4368 | 0.005 | 0.3009 | No |
| 216 | FNTA |  |  | 4443 | 0.003 | 0.2922 | No |
| 217 | LCORL |  |  | 4452 | 0.002 | 0.2913 | No |
| 218 | SNX22 |  |  | 4498 | 0.001 | 0.2860 | No |
| 219 | SASH1 |  |  | 4645 | -0.002 | 0.2688 | No |
| 220 | KIF13A |  |  | 4653 | -0.003 | 0.2680 | No |
| 221 | PKP4 |  |  | 4677 | -0.003 | 0.2653 | No |
| 222 | TMEFF2 |  |  | 4708 | -0.004 | 0.2619 | No |
| 223 | KHSRP |  |  | 4723 | -0.004 | 0.2603 | No |
| 224 | ZCCHC24 |  |  | 4731 | -0.004 | 0.2595 | No |
| 225 | FIBIN |  |  | 4788 | -0.006 | 0.2530 | No |
| 226 | FRMD6 |  |  | 4818 | -0.007 | 0.2497 | No |
| 227 | SIRT2 |  |  | 4877 | -0.008 | 0.2430 | No |
| 228 | PHYHIPL |  |  | 4985 | -0.010 | 0.2306 | No |
| 229 | SEPT8 |  |  | 5032 | -0.012 | 0.2254 | No |
| 230 | PDE1C |  |  | 5040 | -0.012 | 0.2248 | No |
| 231 | SKIL |  |  | 5063 | -0.012 | 0.2224 | No |
| 232 | UIMC1 |  |  | 5130 | -0.014 | 0.2149 | No |
| 233 | RALGPS2 |  |  | 5240 | -0.017 | 0.2023 | No |
| 234 | KLHL5 |  |  | 5313 | -0.019 | 0.1942 | No |
| 235 | TCF7L1 |  |  | 5378 | -0.020 | 0.1870 | No |
| 236 | DOCK1 |  |  | 5496 | -0.024 | 0.1736 | No |
| 237 | CAT |  |  | 5510 | -0.024 | 0.1726 | No |
| 238 | SATB1 |  |  | 5608 | -0.027 | 0.1616 | No |
| 239 | FGF12 |  |  | 5669 | -0.029 | 0.1551 | No |
| 240 | ABI1 |  |  | 5708 | -0.030 | 0.1512 | No |
| 241 | NTM |  |  | 5738 | -0.031 | 0.1484 | No |
| 242 | RAP2C |  |  | 5757 | -0.032 | 0.1469 | No |
| 243 | B9D1 |  |  | 5815 | -0.033 | 0.1409 | No |
| 244 | CDCA7L |  |  | 5899 | -0.036 | 0.1318 | No |
| 245 | CNTN1 |  |  | 5924 | -0.037 | 0.1296 | No |
| 246 | TCF7L2 |  |  | 5935 | -0.037 | 0.1292 | No |
| 247 | CUTC |  |  | 5984 | -0.039 | 0.1243 | No |
| 248 | WASF1 |  |  | 6025 | -0.040 | 0.1204 | No |
| 249 | SH3D19 |  |  | 6053 | -0.041 | 0.1180 | No |
| 250 | DDAH1 |  |  | 6076 | -0.042 | 0.1163 | No |
| 251 | PCMTD2 |  |  | 6084 | -0.042 | 0.1163 | No |
| 252 | ALDH6A1 |  |  | 6109 | -0.043 | 0.1143 | No |
| 253 | GATM |  |  | 6161 | -0.045 | 0.1092 | No |
| 254 | ZC3HAV1 |  |  | 6254 | -0.049 | 0.0993 | No |
| 255 | FBXO7 |  |  | 6283 | -0.049 | 0.0970 | No |
| 256 | BAALC |  |  | 6346 | -0.051 | 0.0907 | No |
| 257 | DENND5A |  |  | 6405 | -0.053 | 0.0849 | No |
| 258 | DUSP6 |  |  | 6502 | -0.056 | 0.0746 | No |
| 259 | PPP2R2B |  |  | 6515 | -0.057 | 0.0744 | No |
| 260 | QKI |  |  | 6517 | -0.057 | 0.0754 | No |
| 261 | CFLAR |  |  | 6582 | -0.060 | 0.0691 | No |
| 262 | NDUFAF2 |  |  | 6588 | -0.060 | 0.0697 | No |
| 263 | TP53INP1 |  |  | 6622 | -0.062 | 0.0670 | No |
| 264 | EVI5 |  |  | 6690 | -0.065 | 0.0604 | No |
| 265 | RSU1 |  |  | 6693 | -0.065 | 0.0615 | No |
| 266 | KCND3 |  |  | 6700 | -0.066 | 0.0621 | No |
| 267 | ARNT2 |  |  | 6737 | -0.068 | 0.0592 | No |
| 268 | C1orf21 |  |  | 6895 | -0.076 | 0.0422 | No |
| 269 | SEZ6L |  |  | 6920 | -0.077 | 0.0409 | No |
| 270 | FYN |  |  | 6924 | -0.077 | 0.0421 | No |
| 271 | CHIC2 |  |  | 6976 | -0.079 | 0.0377 | No |
| 272 | KAT2B |  |  | 7042 | -0.084 | 0.0317 | No |
| 273 | RAP2A |  |  | 7092 | -0.086 | 0.0276 | No |
| 274 | ATP6V0E1 |  |  | 7106 | -0.088 | 0.0278 | No |
| 275 | STARD7 |  |  | 7220 | -0.095 | 0.0164 | No |
| 276 | SNX7 |  |  | 7307 | -0.101 | 0.0083 | No |
| 277 | UBL3 |  |  | 7332 | -0.103 | 0.0075 | No |
| 278 | GUCD1 |  |  | 7419 | -0.110 | -0.0005 | No |
| 279 | CCND1 |  |  | 7456 | -0.113 | -0.0024 | No |
| 280 | SLC25A5 |  |  | 7582 | -0.126 | -0.0147 | No |
| 281 | RAB9A |  |  | 7606 | -0.128 | -0.0148 | No |
| 282 | PRDX3 |  |  | 7649 | -0.132 | -0.0171 | No |
| 283 | CISD1 |  |  | 7700 | -0.139 | -0.0202 | No |
| 284 | ANKS1B |  |  | 7710 | -0.141 | -0.0184 | No |
| 285 | NME1 |  |  | 7811 | -0.154 | -0.0272 | No |
| 286 | TNFRSF21 |  |  | 7822 | -0.155 | -0.0252 | No |
| 287 | ARL4A |  |  | 7829 | -0.155 | -0.0228 | No |
| 288 | CNP |  |  | 7852 | -0.158 | -0.0222 | No |
| 289 | OCIAD2 |  |  | 7906 | -0.167 | -0.0250 | No |
| 290 | PCBP4 |  |  | 8002 | -0.186 | -0.0325 | No |
| 291 | LITAF |  |  | 8015 | -0.188 | -0.0302 | No |
| 292 | DENR |  |  | 8031 | -0.189 | -0.0281 | No |
| 293 | ACP1 |  |  | 8085 | -0.201 | -0.0303 | No |
| 294 | ZMAT3 |  |  | 8086 | -0.201 | -0.0262 | No |
| 295 | SPRY2 |  |  | 8110 | -0.205 | -0.0248 | No |
| 296 | WDR83OS |  |  | 8146 | -0.214 | -0.0246 | No |
| 297 | GLTP |  |  | 8285 | -0.254 | -0.0358 | No |
| 298 | GNG12 |  |  | 8323 | -0.264 | -0.0348 | No |
| 299 | MT3 |  |  | 8329 | -0.265 | -0.0300 | No |
| 300 | NDUFB3 |  |  | 8362 | -0.278 | -0.0282 | No |
| 301 | KHDRBS3 |  |  | 8427 | -0.304 | -0.0296 | No |
| 302 | LYPD1 |  |  | 8460 | -0.321 | -0.0269 | No |
| 303 | RPS27L |  |  | 8528 | -0.351 | -0.0277 | No |
| 304 | DSTN |  |  | 8559 | -0.371 | -0.0237 | No |
| 305 | S100B |  |  | 8562 | -0.373 | -0.0164 | No |
| 306 | DBI |  |  | 8571 | -0.383 | -0.0096 | No |
| 307 | PHLDA1 |  |  | 8670 | -0.476 | -0.0115 | No |
| 308 | RAB31 |  |  | 8696 | -0.514 | -0.0041 | No |
| 309 | FABP5 |  |  | 8699 | -0.518 | 0.0062 | No |
Table: GSEA details [plain text format]

  

Fig 2: RICHARDS\_NOWAKOWSKI\_SCIENCE\_2017\_OPC\_UPREG: Random ES distribution      
 Gene set null distribution of ES for **RICHARDS\_NOWAKOWSKI\_SCIENCE\_2017\_OPC\_UPREG**
